# Supplementary material for: A hyperactivating mutation in formin-like 2 causes brain and vertebrae defects
Source: bioRxiv. 2026 Oct 21:2026.10.16.759550. Preprint. [Version 1] doi: 10.64898/2026.10.16.759550 (PMC13738527; doi:10.64898/2026.10.16.759550)
Supplement: Supplementary file 1 [file NIHPP2026.10.16.759550v1-supplement-1.pdf]

bioRxiv preprint doi: https://doi.org/10.64898/2026.10.16.759550; this version posted October 21, 2026. The copyright holder for this preprint (which was not certified by peer review) is the author/funder, who has granted bioRxiv a license to display the preprint in perpetuity. It is made available under a CC-BY 4.0 International license.

Fig. S1. CRISPR-Cas9-mediated introduction of the *nmf418* mutation recapitulates major phenotypes.

A. Sequencing chromatograms of the *Fmnl2* intron 9/exon 10 boundaries in wild-type (WT), *418*$^{-/-}$, and *Fmnl2*$^{In9A>C/In9A>C}$ genomic DNA. Arrow indicates the A>C transversion at the canonical AG splice acceptor site of intron 9. The same WT and *418*$^{-/-}$ chromatograms are shown in Fig 2C. **B.** Midline sagittal sections of P21 WT and *Fmnl2*$^{In9A>C/In9A>C}$ cerebella stained with hematoxylin and eosin, demonstrating cerebellar hypoplasia and lobule disorganization in *Fmnl2*$^{In9A>C/In9A>C}$ mice. Scale bar = 200µm. **C.** Representative western blot for FMNL2 in cortex, cerebellum (Cb), hippocampus (Hipp), and lung from WT and *Fmnl2*$^{In9A>C/In9A>C}$ mice, demonstrating a dramatic reduction in FMNL2 protein levels. HSP90 was used as a loading control.

bioRxiv preprint doi: https://doi.org/10.64898/2026.10.16.759550; this version posted October 21, 2026. The copyright holder for this preprint (which was not certified by peer review) is the author/funder, who has granted bioRxiv a license to display the preprint in perpetuity. It is made available under a CC-BY 4.0 International license.

Fig. S2. Exogenous FMNL2Δ25 promotes increased actin filamentation when accounting for protein levels.

A. Representative western blot for eGFP and actin in input lysates from HEK293T cells (Fig. 3C, D) overexpressing eGFP, wild-type FMNL2-eGFP, or FMNL2Δ25-eGFP prior to globular (G) and filamentous (F) actin fractionation. Note the reduced expression of FMNL2Δ25-eGFP relative to wild-type FMNL2-eGFP. Input samples were available from two of the three fractionated replicates. **B.** F:G actin ratio differences from Fig. 3D normalized to the level of exogenous eGFP-tagged protein from Panel A, demonstrating an approximately 5-fold greater ratio in FMNL2Δ25-eGFP-expressing cells relative to those expressing wild-type FMNL2-eGFP.

bioRxiv preprint doi: https://doi.org/10.64898/2026.10.16.759550; this version posted October 21, 2026. The copyright holder for this preprint (which was not certified by peer review) is the author/funder, who has granted bioRxiv a license to display the preprint in perpetuity. It is made available under a CC-BY 4.0 International license.

Fig. S3: Schematic of the SRE-firefly luciferase reporter assay.

Under basal conditions (left), MAL (MRTF-A) is sequestered in the cytoplasm by free G-actin, preventing serum response factor (SRF) from binding the serum response element (SRE) and driving firefly luciferase expression. Upon actin polymerization (right), the free G-actin pool is reduced, MAL translocates to the nucleus, binds SRF at the SRE, and drives firefly luciferase accumulation, providing an indirect readout of upstream actin polymerization activity.

bioRxiv preprint doi: https://doi.org/10.64898/2026.10.16.759550; this version posted October 21, 2026. The copyright holder for this preprint (which was not certified by peer review) is the author/funder, who has granted bioRxiv a license to display the preprint in perpetuity. It is made available under a CC-BY 4.0 International license.

Fig. S4: Postnatal lethality in *Fmnl2$^{418/418}$* but not *Fmnl2$^{-/-}$* mice.

A. Schematic of the conditional *Fmnl2* knockout allele. LoxP sites were inserted flanking the 77-bp exon 4 to generate *Fmnl2$^{fl/fl}$* mice. Sox2-Cre-mediated recombination deletes exon 4, causing a frameshift and premature stop codon, producing a null *Fmnl2$^{-/-}$* allele. **B.** Representative western blot for FMNL2 in cerebellar extracts from WT and *Fmnl2$^{-/-}$* mice confirming complete loss of FMNL2 protein following exon 4 deletion by Sox2-Cre. HSP90 was used as a loading control. **C.** Observed proportions of E18.5 and/or P21 progeny from intercrosses of *Fmnl2$^{+/418}$*, *Fmnl2$^{+/In9A>C}$*, or *Fmnl2$^{+/-}$* heterozygous mice. *Fmnl2$^{418/418}$* and *Fmnl2$^{In9A>C/In9A>C}$* homozygotes are present at Mendelian ratios at E18.5 but are underrepresented at P21 indicating postnatal lethality. *Fmnl2$^{-/-}$* mice are recovered at normal Mendelian ratios at P21. Statistical significance was assessed by Chi-square test.

bioRxiv preprint doi: https://doi.org/10.64898/2026.10.16.759550; this version posted October 21, 2026. The copyright holder for this preprint (which was not certified by peer review) is the author/funder, who has granted bioRxiv a license to display the preprint in perpetuity. It is made available under a CC-BY 4.0 International license.

Fig. S5. *Nmf418* phenotypes require two mutant alleles.

A. Hematoxylin and eosin-stained coronal sections of E11.5 wild-type (WT) and *Fmnl2*$^{418/418}$ (*418*$^{-/-}$) embryos at anterior to posterior (left to right) levels of the neural tube. Higher magnification images of the dorsal region of the neural tube (boxed) are shown below. The neural tube is closed across the entire anterior-posterior axis in *Fmnl2*$^{418/418}$ embryos, indicating that the spinal column opening is not caused by failure of neural tube closure. Top Scale bars = 100 µm (top) and 40µm (bottom). **B.** Transverse lumbar spinal sections of E18.5 WT and *Fmnl2*$^{418/418}$ mice stained with cresyl violet. Notice the flattening of the spinal cord and lack of fusion of the spinous process in the mutant. Scale bar = 200µm. **C.** Dorsal views of alcian blue (cartilage)/alizarin red (bone)-stained skeletons from WT and *Fmnl2*$^{+/418}$ (*418*$^{+/-}$) E18.5 embryos. **D.** Midline sagittal sections of cerebella from E18.5 WT and *Fmnl2*$^{+/418}$ mice. Asterisks denote visible principal fissures. pc, preculminate fissure; pr, primary fissure; sec, secondary fissure; pl, posterolateral fissure. Scale bar = 100µm. **E.** Transverse lumbar spinal sections of E18.5 WT and *Fmnl2*$^{+/418}$ mice stained with cresyl violet. Scale bar = 200µm. **F.** Caudal views of cerebella from P21 WT and *Fmnl2*$^{+/418}$ mice. SC = superior colliculus; IC = inferior colliculus; H = hemisphere; V = vermis. **G.** Midline sagittal sections of P21 WT and Fmnl2$^{418/-}$ cerebella stained with hematoxylin and eosin. Lobules are indicated by Roman numerals. Scale bar = 200µm. **H.** Observed proportions of P21 progeny from a cross between *Fmnl2*$^{+/418}$ and *Fmnl2*$^{-/-}$ mice. Statistical significance was assessed by Chi-square test.

bioRxiv preprint doi: https://doi.org/10.64898/2026.10.16.759550; this version posted October 21, 2026. The copyright holder for this preprint (which was not certified by peer review) is the author/funder, who has granted bioRxiv a license to display the preprint in perpetuity. It is made available under a CC-BY 4.0 International license.

Fig. S6: *Fmnl2* deletion or mutation do not alter cell proliferation in mouse embryonic fibroblasts.

A. Representative western blot for FMNL2 in mouse embryonic fibroblasts (MEFs) derived from WT, *Fmnl2*$^{418/418}$, and *Fmnl2*$^{-/-}$ embryos. FMNL2 is markedly reduced in *Fmnl2*$^{418/418}$ MEFs and absent in *Fmnl2*$^{-/-}$ MEFs. GAPDH was used as a loading control. **B, C.** Representative Incucyte images of WT and *Fmnl2*$^{-/-}$ MEF (**B**) and WT and *Fmnl2*$^{418/418}$ (**C**) wound healing assays at 0, 12, and 24 hours, corresponding to the quantification in Figure 4H. Scale bars = 100µm. **D.** Quantification of the percentage of WT, *Fmnl2*$^{418/418}$, and *Fmnl2*$^{-/-}$ MEFs positive for BrdU (S-phase), Ki67 (actively cycling), both BrdU and Ki67, or Ki67 alone. Data are means ± SEM. No significant differences in proliferative status were detected across genotypes by two-way ANOVA with Tukey's multiple comparisons test, confirming that differences in wound closure reflect cell motility rather than proliferation.

bioRxiv preprint doi: https://doi.org/10.64898/2026.10.16.759550; this version posted October 21, 2026. The copyright holder for this preprint (which was not certified by peer review) is the author/funder, who has granted bioRxiv a license to display the preprint in perpetuity. It is made available under a CC-BY 4.0 International license.

Figure S7: Differential expression analysis and clustering of *Fmnl2*$^{418/418}$ and *Fmnl2*$^{-/-}$ cerebella.

A. Volcano plots depicting the spread of gene expression changes between wild-type (WT) and *nmf418*$^{-/-}$ or *Fmnl2*$^{-/-}$ groups. Differentially expressed genes (p-adj < 0.05) are highlighted in blue if downregulated and red if upregulated in the respective comparisons. **B.** Venn diagrams of the number of upregulated (top) and downregulated (bottom) genes that overlapped between *Fmnl2*$^{418/418}$ (*418*$^{-/-}$) and *Fmnl2*$^{-/-}$ cerebella when compared to wild-type. **C.** Principal component analysis (PCA) plot of RNA sequencing results depicts clustering of samples by genotype.

Figure S1

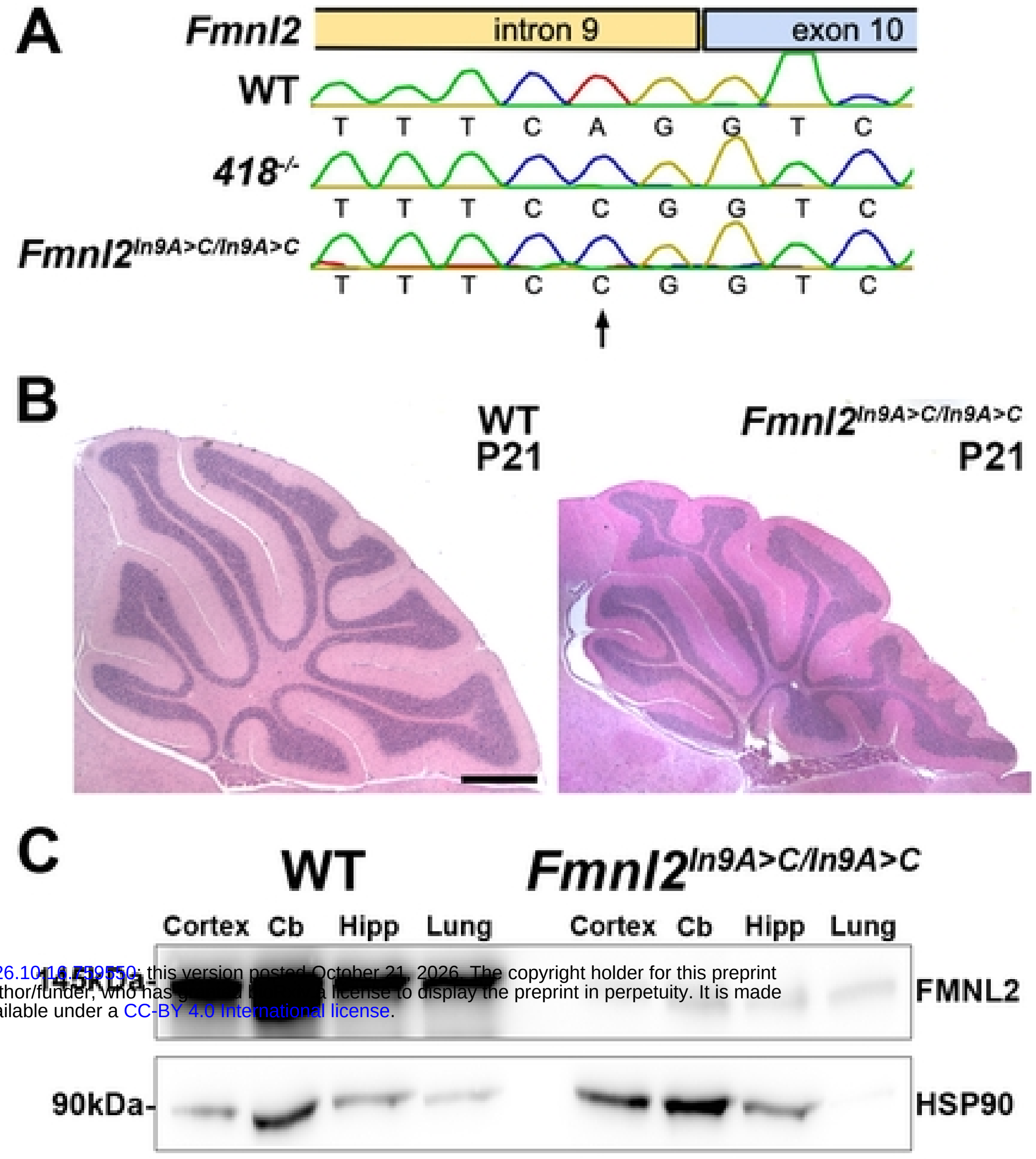

bioRxiv preprint doi: https://doi.org/10.64898/2026.10.18.682850; this version posted October 21, 2026. The copyright holder for this preprint (which was not certified by peer review) is the author/funder, who has granted bioRxiv a license to display the preprint in perpetuity. It is made available under a CC-BY 4.0 International license.

Figure S1

Figure S2

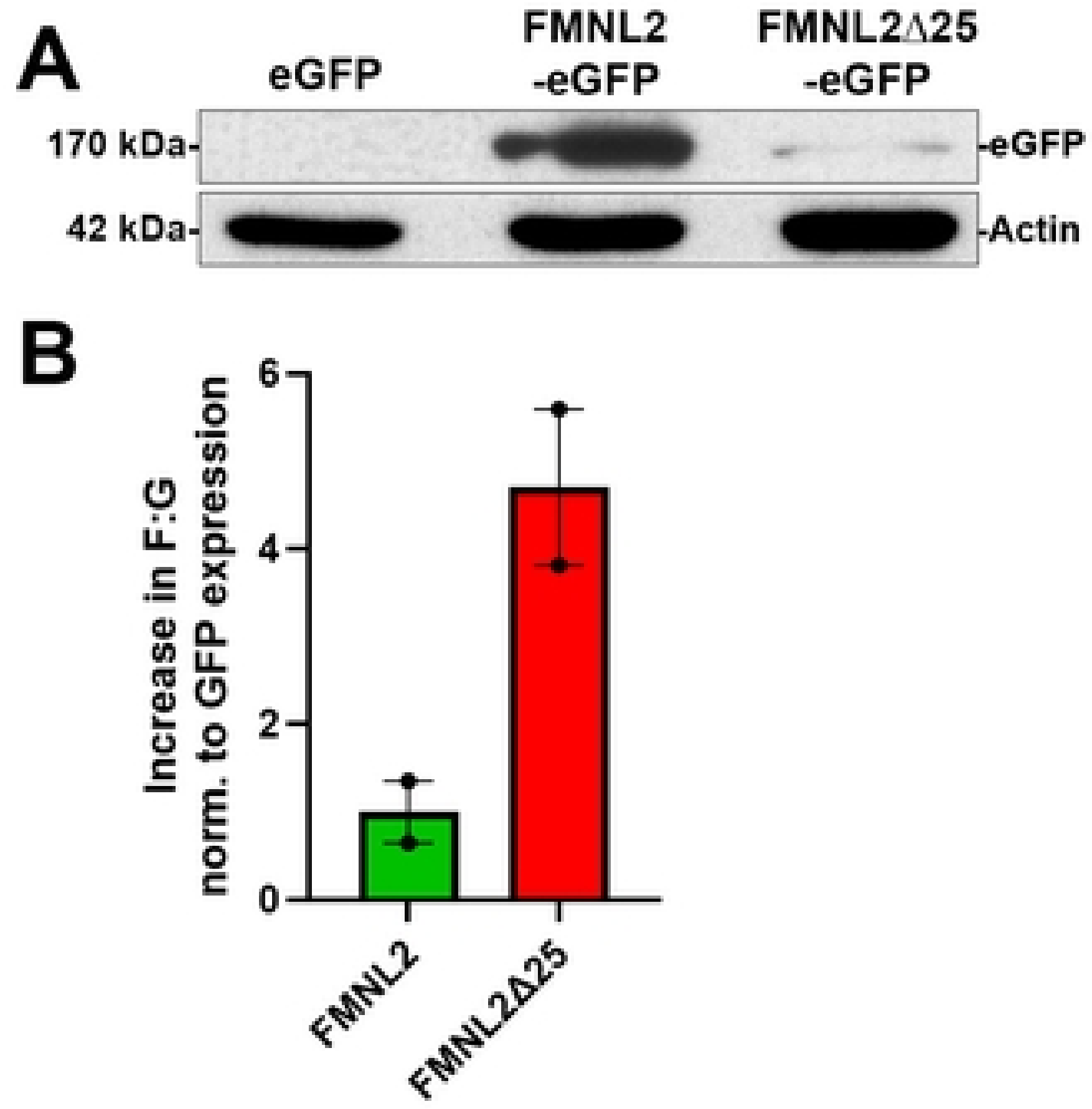

bioRxiv preprint doi: https://doi.org/10.64898/2026.10.16.759550; this version posted October 21, 2026. The copyright holder for this preprint (which was not certified by peer review) is the author/funder, who has granted bioRxiv a license to display the preprint in perpetuity. It is made available under a CC-BY 4.0 International license.

Figure S2

Figure S3

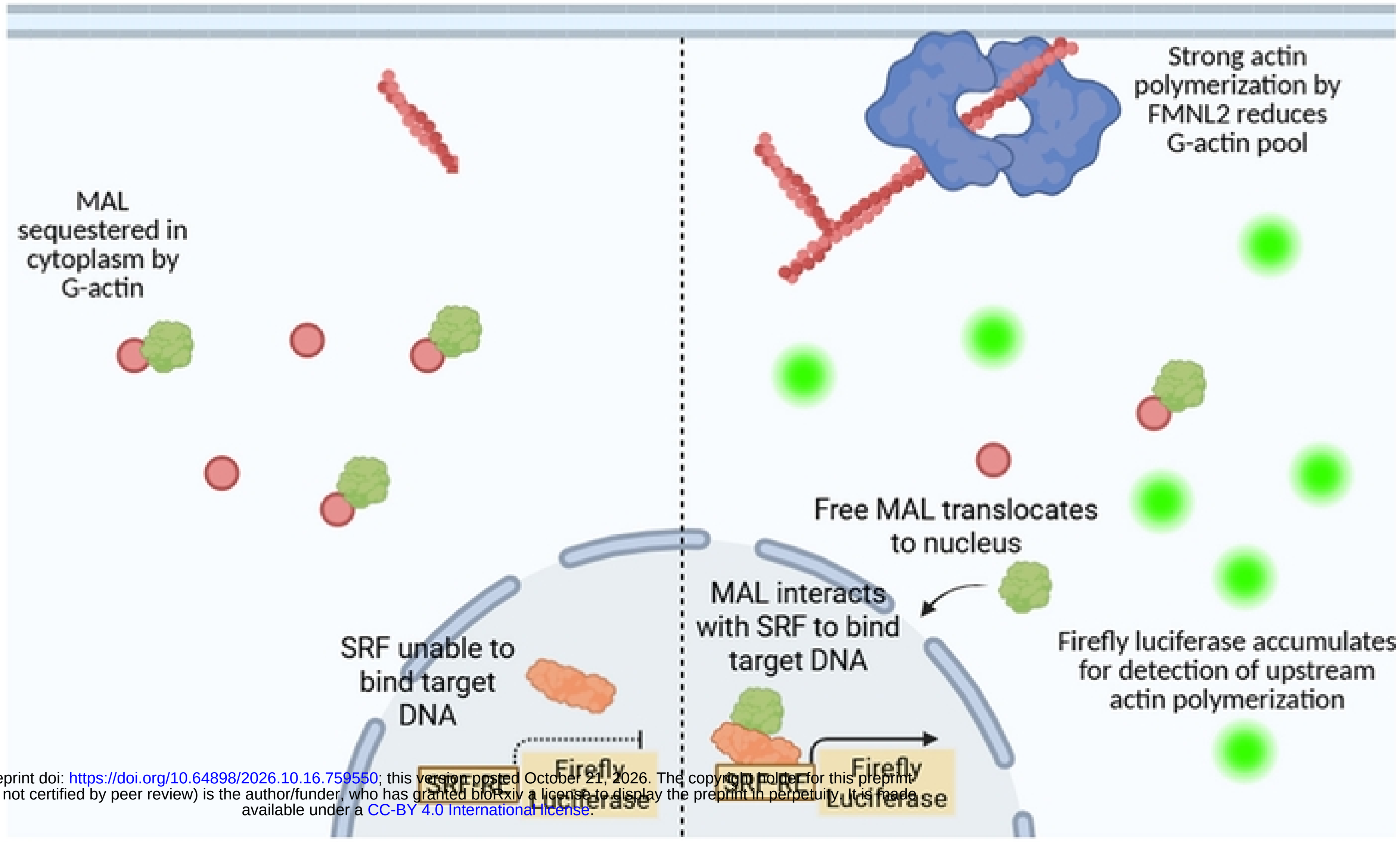

bioRxiv preprint doi: https://doi.org/10.64898/2026.10.16.759550; this version posted October 21, 2026. The copyright holder for this preprint (which was not certified by peer review) is the author/funder, who has granted bioRxiv a license to display the preprint in perpetuity. It is made available under a CC-BY 4.0 International license.

Figure S3

Figure S4

A B

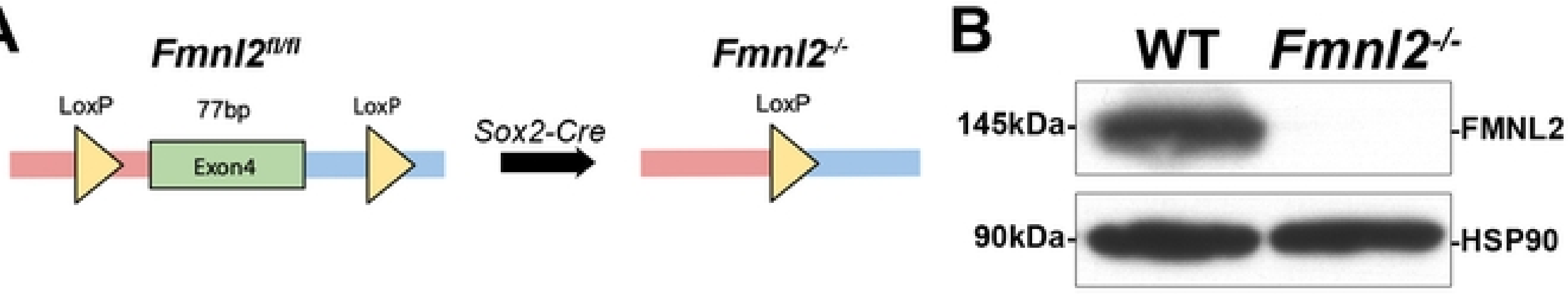

C

	WT		*Fmnl2*$^{+/418}$		*Fmnl2*$^{418/418}$		
Age	Number	%	Number	%	Number	%	Chi-Square p-value
E18.5	30	27.78	56	51.85	22	20.37	0.5134
P21	106	37.06	169	59.09	11	3.85	<0.0001

	WT		*Fmnl2*$^{+/In9A>C}$		*Fmnl2*$^{In9A>C/In9A>C}$		
Age	Number	%	Number	%	Number	%	Chi-Square p-value
P21	141	44.62	163	51.58	12	3.80	<0.0001

	WT		*Fmnl2*$^{+/-}$		*Fmnl2*$^{-/-}$		
Age	Number	%	Number	%	Number	%	Chi-Square p-value
P21	45	23.68	105	55.26	40	21.05	0.306

bioRxiv preprint doi: https://doi.org/10.64898/2026.10.16.759550; this version posted October 21, 2026. The copyright holder for this preprint (which was not certified by peer review) is the author/funder, who has granted bioRxiv a license to display the preprint in perpetuity. It is made available under a CC-BY 4.0 International license.

Figure S4

Figure S5

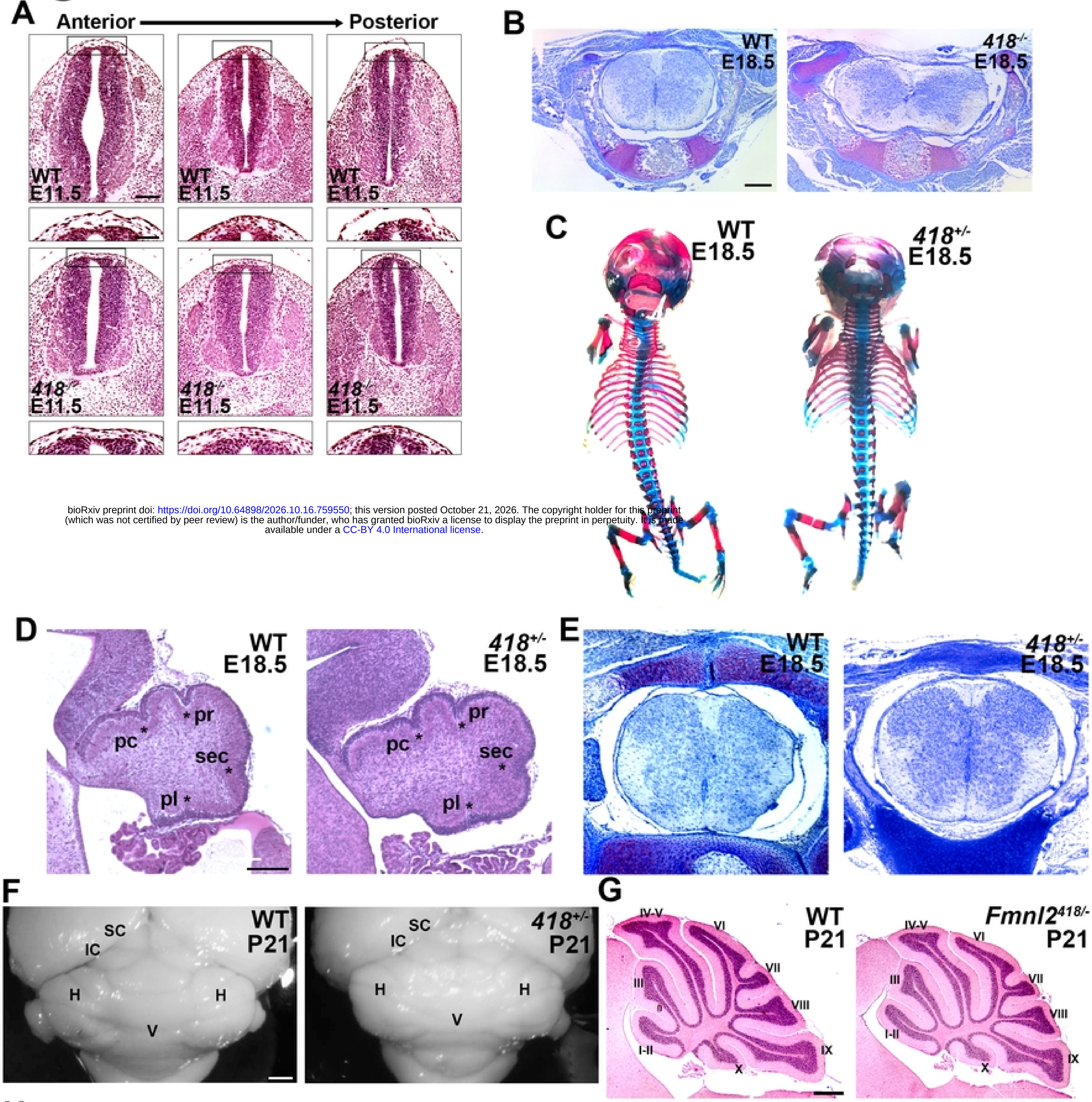

bioRxiv preprint doi: https://doi.org/10.64898/2026.10.16.759550; this version posted October 21, 2026. The copyright holder for this preprint (which was not certified by peer review) is the author/funder, who has granted bioRxiv a license to display the preprint in perpetuity. It is made available under a CC-BY 4.0 International license.

H

	Fmnl2$^{+/-}$		*Fmnl2*$^{418/-}$		
Age	Number	%	Number	%	Chi-Square p-value
P21	8	42.11	11	57.89	0.4913

Figure S5

Figure S6

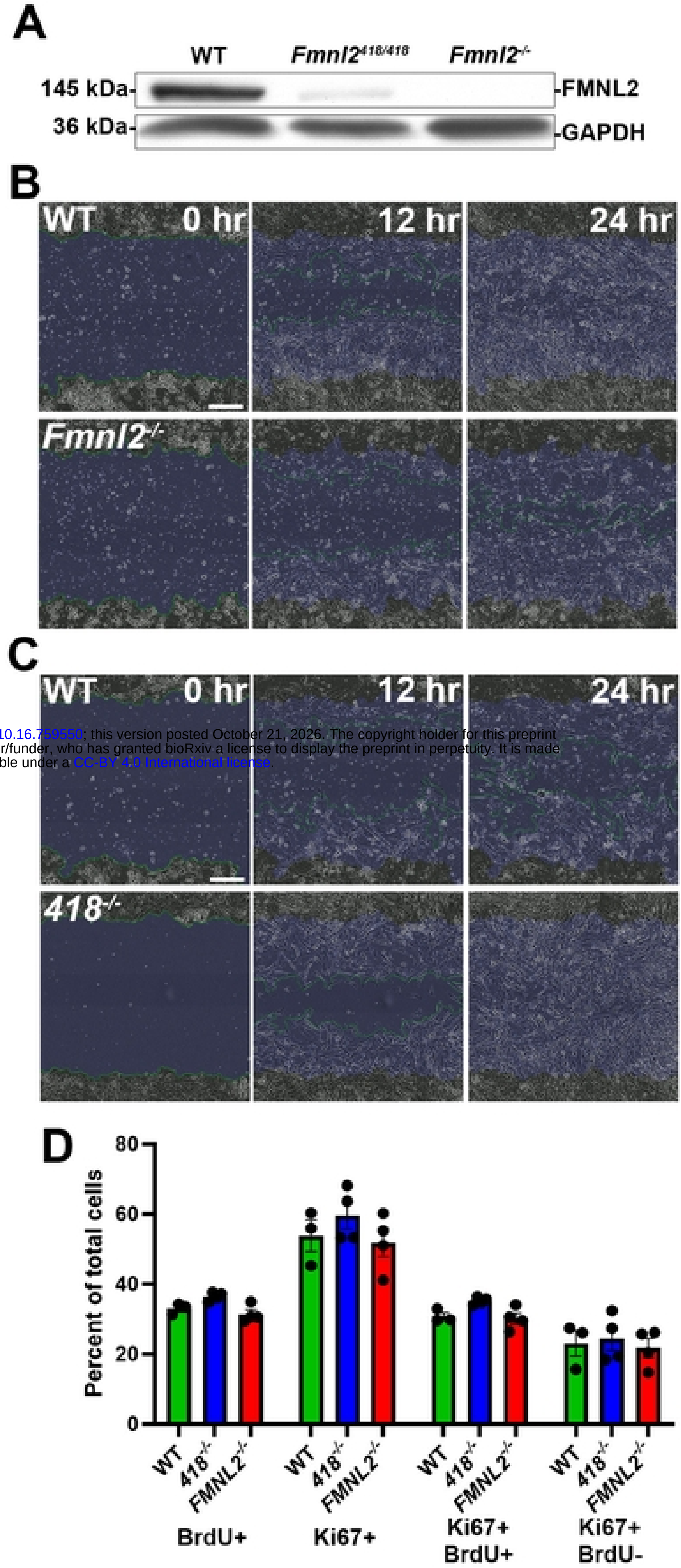

bioRxiv preprint doi: https://doi.org/10.64898/2026.10.16.759550; this version posted October 21, 2026. The copyright holder for this preprint (which was not certified by peer review) is the author/funder, who has granted bioRxiv a license to display the preprint in perpetuity. It is made available under a CC-BY 4.0 International license.

Figure S6

Figure S7

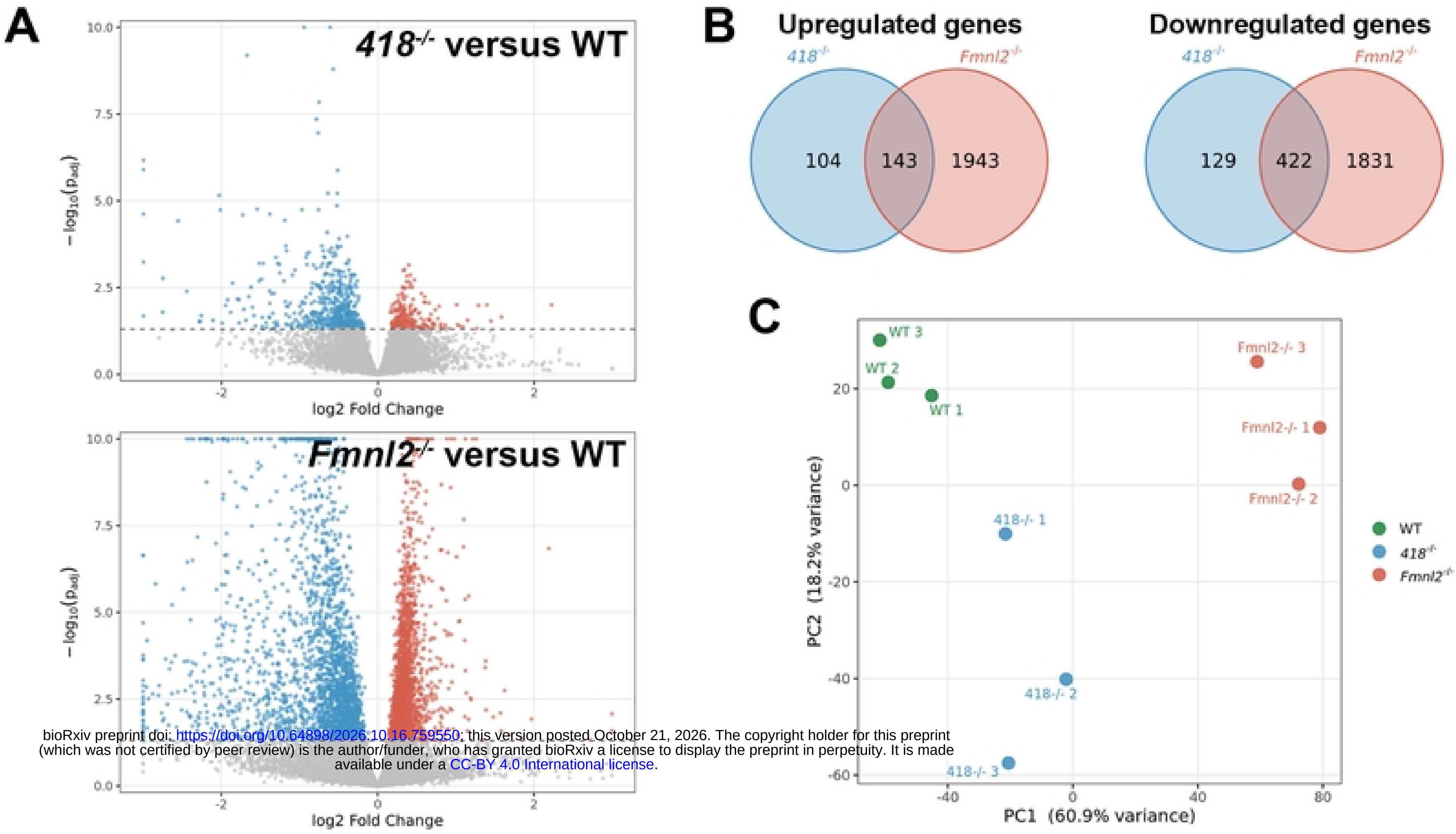

bioRxiv preprint doi: https://doi.org/10.64898/2026.10.16.759550; this version posted October 21, 2026. The copyright holder for this preprint (which was not certified by peer review) is the author/funder, who has granted bioRxiv a license to display the preprint in perpetuity. It is made available under a CC-BY 4.0 International license.

Figure S7
